# A New Method for Rapidly Uniting Wounds by First Intention

**Published:** 1848-04

**Authors:** 


### *A New Method for rapidly uniting Wounds by first intention.*

[Communicated for the Boston Medical and Surgical Journal]

It is well known that common cotton, subjected for a certain length of time to the action of nitric and sulphuric acids, combined in stated proportions, is so changed in its intimate structure as to acquire an explosive property.

Professor Schonbein originally demonstrated this discovery, and ascertained the fact that prepared in a certain manner, this cotton is capable of solution in sulphuric ether.* It is known in the community by a name acquired from its explosive quality—*gun* cotton. I learned the manner of preparing this cotton, and of dissolving it in ether, from Dr. Chas. T. Jackson, who remarked upon it and exhibited specimens before the Natural History Society, in Dec. 1846, or Jan. 1847. He enumerated various uses to which it might be applied—among others, for a brilliant varnish. For this use I soon after prepared a bottle, according to his directions. While engaged in employing it in this way, I accidently smeared with it a fresh wound on my finger. The smarting called my attention to it, and I endeavored immediately to rub it off. It had dried, however, instantaneously, and remained on. The very soon ceased, and when the film was removed, perfect union had taken place. Since this time I have been testing the efficacy of this preparation, as opportunities have occurred, as a dressing for wounds, especially those which it is desirable to unite rapidly, by first intention. It will be seen to possess, very eminently, all the requirements for producing such a union.

1st. By its powerful contraction, upon evaporation, it places the edges of incised wound in much more intimate contact than is obtained by sutures and adhesive cloth—unites them by equal pressure throughout the whole extent of wound, and maintains them immovably fixed.

---

* It has been shown to be soluble in chloroform.

2d. It preserves the wound perfectly from contact with the air—being impermeable to the atmosphere, while its adhesion to the skin is so intimate as to preclude the possibility of the air entering beneath its edges.

3d. The substances remaining in contact with the skin and wound after the evaporation of the ether, seems to be entirely inert so far as any irritating property is concerned, and this can hardly be said of any resinous adhesive cloth or preparation.

4th. It does away with the necessity for sutures in incised wounds of almost any extent.

5th, It is sure to remain in intimate contact with the skin until union is complete—and being quite impervious to water, and presenting a polished surface, it allows the surrounding parts to be washed without regard to the wound or dressing.

6th. It is colorless and transparent, thus permitting the surgeon to witness all that goes on beneath, without involving the necessity for its removal.

7th. No heat is necessary for its application, and the presence of any moderate degree of cold is only objectionable in retarding the evaporation of the ether.

8th. It may be made at a trifling cost—an ounce phial, intrinsically worth little, being sufficient for a great number of dressings.

It is not incised wounds alone which are amenable to its use, though the mode of its application to a stump, or an ulcer, or any wound involving an extensive loss of skin, must be modified.

It is of the first importance that this preparation be properly made and applied. The process for the application is very simple.

For straight incisions of *whatever length*, provided the edges can be brought together without great difficulty, it is better to apply the solution in immediate contact with the skin—as follows:—The bleeding should be arrested, and skin thoroughly dried, If the lips of the wound are themselves in contact, the surgeon has only to apply a coating of the solution lengthwise over the approximated edges by means of a camel's hair pencil, leaving it untouched after the brush has once passed over it till it is dry, during, perhaps, ten or twenty seconds. This first film will of itself have continued the edges together; but in order to increase the firmness of the support, more must then be applied in the same manner, allowing it to extend on either side of the incision a half an inch or more. If however the wound gapes, an assistant is required to bring the edges in contact and retain them so whilst the application is made. If the incision is so long that the assistant cannot place the edges in apposition throughout the whole extent, begin by covering a small portion at the upper end, and apply the solution to the lower parts as fast as it becomes dry above.* In this case something more

* Having made a dog insensible with ether, I made an incision down the back where the hair had been removed by an old scald six or eight inches in length, and dressed it alone with the preparation, without a suture. The union was perfect the whole extent n about thirty hours, even in the old cicatrix.

than the film which is left adherent to the skin will be necessary for a safe and proper support to the wound, which may have a tendency to separate. The transparency of the dressing may be still maintained by adapting a piece of gold-beater's skin or oiled silk to the wound. This should be covered with the solution, and the membrane applied after the coating is on and already contracted. A dossil of lint, or a strip of cloth, or even a piece of tissue paper which is thus rendered tough and water-proof, will answer the same purpose, though not transparent. Where there is much separation, it is better to fortify the wound in this way at once, and as fast as the first coating is applied and dry.

In dressing the wound left by the removal of the breast, the preparation may be applied in the same way. If, however, adhesion by first intention be not desired, the gum may be painted on in transverse strips, like adhesive cloth, letting the first strip dry and giving it the gold-beater's skin support before the second is applied. Thus room is left for the escape of pus, and the exposed portion may be watched without removing the strips.

As a dressing after the operation for hare-lip or cancer of the lip, where union by first intention and a narrow linear cicatrix are so desirable, this answers particularly well. The use of one or two sutures to the mucous surface is not obviated, as the solution will not adhere to the moist epithelium, or to a surface secreting mucus, with sufficient certainty. But this does not interfere at all with the satisfactory result upon the cuticle, as the skin will be probably united before the necessity for removing the sutures arrives.

In operations for the restoration of parts, as, for instance, the nose, where union by first intention is important, we have had no opportunity to see it applied, but from analogy do not doubt that it would succeed perfectly, as it fulfils so entirely many of the requirements for such union. The same of all plastic operations; and a drop placed upon a small cut, or the puncture of a sub-cutaneous operation, seals them hermetically.

In dressing an ulcer, where there is, of course, a loss of soft parts, it is better to apply it through the intervention of some medium. Let a strip of cloth or gold-beater's skin be cut of sufficient length, then let the two ends be covered thickly an inch or more, with the solution. Apply this strip, like a strip of adhesive cloth, so that the middle of the cloth, where there is none of the solution, shall come over the ulcer. After all the strips are applied, the air may be excluded by painting the cloth upon the outside over the ulcer with the solution. The same contraction goes on in drying, and so approximates the edges of the ulcer, and gives it firm support.

These are a few points which may serve to illustrate the general plan of the application of the adhesive gum to wounds—it must be left to the surgeon to make special investigation, as particular case may demand.

To anticipate an obvious objection; the momentary pain arising from the direct application of the ether to an incised surface, may be in a great measure prevented by the intimate apposition of the edges of the wound. Again, this stimulus in brief, and probably more than counteracted by the refrigeration influence of the evaporating ether. There are undoubtedly cases when such a stimulus would prove beneficial. It is even possible that the rapidity of the union which takes place under a coating of this gum, may be due, in part, to the influence of this stimulus.

I will allude, in a few words, to some of the surgical uses of the solution of gun cotton unconnected with the dressing of wounds. It may probably be applied instead of starch to a bandage enveloping a limb. Here, again, its power of contraction is a desideratum, as a snug casing is generally desired, and the force is exerted equally. Perhaps the limb may be immersed in the solution without the intervention of the bandage: Several coatings will here be required. Its use as a means of rendering pasteboard splints impervious to water has been suggested to me by Dr. H. J. Bigelow; and a hundred other applications may be made of it at the bedside by the surgeon, who knows its nature and qualities. The pathologist, with his abrasions thus protected, may enter the inflamed peritoneal cavity with impunity, or examine fearlessly the products of inoculable lesions. In dissection, hang-nails, sores, or abrasions of any kind, will be thus fully protected.

I am informed that a series of experiments are being now made at the Massachusetts General Hospital, by the surgeons in attendance, who will be soon able to test its value and range of application.

BOSTON, March 16, 1848. S. L. BIGELOW.

